# Manual of Gynecology

**Published:** 1898-12

**Authors:** 


Manual of Gynecology. By Henry T. Byford, M. D., Professor of Gynecology and Clinical Gynecology in the College of Physicians and Surgeons; Professor of Clinical Gynecology in the Medical Department of Northwestern University; Professor of Gynecology in the Post Graduate Medical School of Chicago. Second Edition. Illustrated. Published by P. Blakiston, Son & Co., Philadelphia. 1897. Pages, 595. Price, cloth, $3.

The author is well known as one of our leading authorities and the present work in its first edition is familiar to all students. This, the second edition, is an improvement in many respects. A large amount of new matter has been added without increasing the scope or aims of the work, which is particularly addressed to students. In the present edition, new "Parts" on carcinoma, sarcoma, cystic tumors and on the anatomy of the pelvic organs have been inserted, as well as new chapters upon venereal diseases. Many of the chapters have been entirely rewritten and the work is fully brought up to date.

The work in its present form is one of the best text-books for students and is strongly recommended. I. J. J.

